# Metabolically healthy obesity increases the risks of MASLD and hyperuricemia: a cohort study with mediation analysis

**DOI:** 10.1186/s12902-025-02106-9

**Published:** 2025-11-27

**Authors:** Yiyun Liu, Tianchen Qian, Yiyang Zheng, Aiming Liu, Lei Xu, Guoliang Ye, Jiarong Xie

**Affiliations:** 1 Department of Gastroenterology, The First Affiliated Hospital of Ningbo University, No.59. Liuting Street, Haishu District, Ningbo, 315000 China; 2 Health Science Center, Ningbo University, Ningbo, Zhejiang 315211 China; 3 Department of Gastroenterology, The First Affiliated Hospital, College of Medicine, Zhejiang University, Hangzhou, 310003 China

**Keywords:** Metabolically healthy obesity, MASLD, Hyperuricemia, Mediation analysis, Metabolic indicators

## Abstract

**Background:**

Although metabolically healthy obesity (MHO) is often regarded as a relatively benign phenotype, its association with the risk of metabolic dysfunction-associated steatotic liver disease (MASLD) and hyperuricemia remains unclear. This study examined the associations between MHO and other metabolic-obesity phenotypes with MASLD and hyperuricemia, and explored the mediating roles of metabolic indicators.

**Methods:**

This study included 11,712 and 13,846 participants from a health examination cohort at the First Affiliated Hospital of Ningbo University for MASLD and hyperuricemia analyses, respectively. Participants were classified into four metabolic-obesity phenotypes, with MHO defined as obesity without metabolic syndrome components. Cox regression and mediation analyses were performed to examine the associations and the proportions explained by metabolic indicators.

**Results:**

During a 7-year follow-up period, 3,756 MASLD and 3,294 hyperuricemia cases were identified. Compared with the metabolically healthy non-obese group, individuals with MHO exhibited significantly higher risks of MASLD (hazard ratio [HR] = 3.54, 95% confidence interval [CI]: 3.09–4.05) and hyperuricemia (HR = 1.92, 95% CI: 1.65–2.23). Mediation analysis illustrated that the triglyceride glucose index accounted for 16.2% and 13.8% of the total effects, while other indicators also contributed to the associations to varying degrees.

**Conclusion:**

MHO is associated with an increased risk of MASLD and hyperuricemia, partly explained by metabolic indicators. Greater attention to these indicators may facilitate earlier risk identification and more effective prevention.

**Clinical trial registration:**

NCT07001865; Registration date: May 25, 2025; ClinicalTrials.gov.

**Supplementary information:**

The online version contains supplementary material available at 10.1186/s12902-025-02106-9.

## Introduction

Metabolic dysfunction-associated steatotic liver disease (MASLD) is currently the most prevalent chronic liver disease worldwide, affecting approximately 38% of the general adult population [1]. As a hepatic manifestation of metabolic syndrome (MetS), MASLD is associated with obesity, insulin resistance (IR), dyslipidemia, type 2 diabetes mellitus, and cardiovascular diseases [2]. Similarly, hyperuricemia, characterized by elevated serum uric acid levels due to abnormal purine metabolism, shares similar metabolic risk factors, including MetS, obesity, and IR [3, 4]. The increasing prevalence of both MASLD and hyperuricemia poses a growing challenge to global public health systems [5].

Recently, metabolic-obesity phenotypes have been proposed to distinguish individuals based on body mass index (BMI) and metabolic health status [6]. Among these phenotypes, metabolically healthy obesity (MHO) describes individuals with obesity who do not meet the diagnostic criteria for MetS [7]. Although MHO has been considered as a relatively benign phenotype, emerging evidence indicates its association with an increased risk of type 2 diabetes mellitus and cardiovascular disease compared to metabolically healthy individuals of normal weight. These risks may be linked to IR and lipid accumulation, contributing to MASLD and hyperuricemia development [8, 9]. However, the long-term metabolic implications of MHO for these conditions remain unclear, and the mediating role of metabolic indicators beyond conventional MetS components remains incompletely understood.

Consequently, this cohort study aimed to assess the associations of different metabolic-obesity phenotypes with the risks of MASLD and hyperuricemia and to explore the mediating effects of metabolic indicators that are not included in the current definitions of metabolic health. These findings may provide new insights into the underlying metabolic risks of MHO and support improved risk stratification in this population.

## Materials and methods

### Study population

**Fig. 1:**
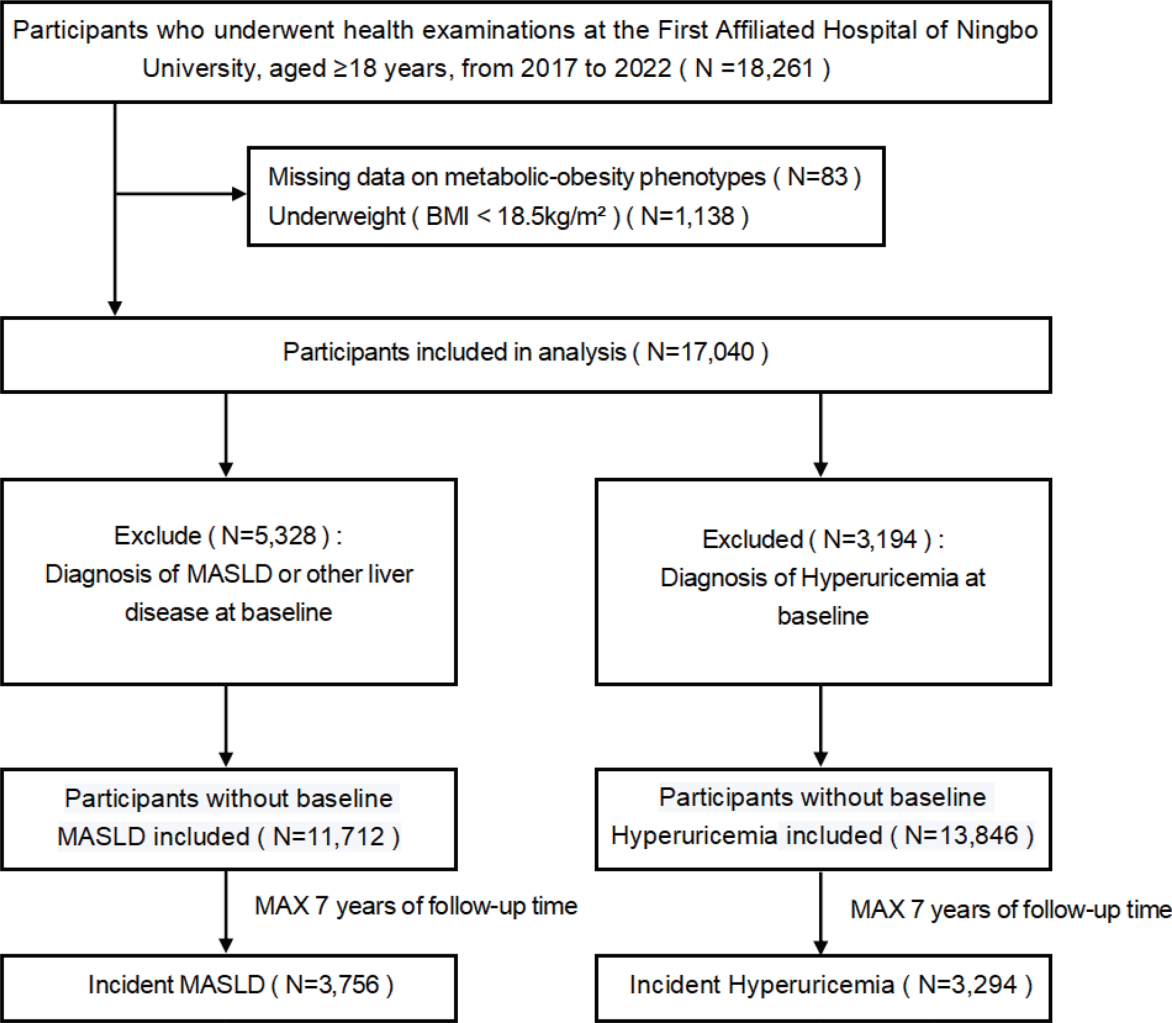
Flowchart of the study participants

This cohort study included participants who underwent comprehensive health examinations at the Physical Examination Center of the First Affiliated Hospital of Ningbo University, where most outpatient visits and laboratory tests were conducted within the same hospital system. Baseline examinations were conducted between January 1, 2017, and November 30, 2022. Initially, participants aged ≥ 18 years who had undergone abdominal ultrasound examinations were included (*N* = 18,261). According to the inclusion and exclusion criteria, participants who were underweight (BMI < 18.5 kg/m^2^; *N* = 1,138) or had missing information on MetS components (*N* = 83) were excluded, leaving 17,040 participants for further analysis. Subsequently, individuals diagnosed with MASLD or other liver diseases at baseline were also excluded (*N* = 5,328), leaving 11,712 participants eligible for the MASLD incidence analysis. In parallel, those diagnosed with hyperuricemia at baseline were excluded (*N* = 3,194), leaving 13,846 participants eligible for the hyperuricemia incidence analysis. Participants were followed from the baseline examination date to the first diagnosis of MASLD or hyperuricemia or the last available examination before July 1, 2024, whichever occurred first. Person-years were calculated as the time between the baseline and censoring dates. During the 7-year follow-up period, incident cases of MASLD (*N* = 3,756) and hyperuricemia (*N* = 3,294) were documented (Fig. 1).

### Assessment of metabolic-obesity phenotypes

Obesity was classified based on BMI, calculated as body weight divided by height (kg/m^2^). Based on the cutoff values indicated by the Western Pacific Regional Office of the World Health Organization [10], obesity was defined as a BMI ≥ 25.0 kg/m^2^. Metabolic health was defined based on the criteria proposed by Ortega et al. [11]. Participants were classified as metabolically healthy if they did not meet any of the following four components of MetS at baseline: (1) systolic blood pressure ≥ 130 mmHg or diastolic blood pressure ≥ 85 mmHg; (2) fasting blood glucose (FBG) ≥ 5.6 mmol/L; (3) high-density lipoprotein cholesterol (HDL-C) < 1.0 mmol/L for male or < 1.3 mmol/L for female; and (4) triglycerides (TG) ≥ 1.7 mmol/L. Those meeting one or more of these criteria were classified as metabolically unhealthy. Based on BMI and metabolic health status, participants were categorized into four distinct metabolic-obesity phenotypes: (1) Metabolically healthy non-obese (MHNO); (2) metabolically unhealthy non-obese (MUNO); (3) metabolically healthy obese (MHO); and (4) metabolically unhealthy obese (MUO).

### Definition of MASLD and hyperuricemia

MASLD was defined as hepatic steatosis detected by ultrasonography, with the presence of at least one of the following cardio-metabolic risk factors and in the absence of excessive alcohol consumption ( > 30 g/day for male or > 20 g/day for female) [12]: (1) BMI ≥ 23 kg/m^2^ or waist circumference > 90 cm in male and > 80 cm in female; (2) FBG ≥ 5.6 mmol/L or glycated hemoglobin ≥ 5.7%; (3) Blood pressure ≥ 130/85 mmHg; (4) TG ≥ 1.7 mmol/L; (5) HDL-C ≤ 1.0 mmol/L in male and ≤ 1.3 mmol/L in female. Hyperuricemia was defined as uric acid (UA) level ≥ 420 µmol/L in male and ≥ 360 µmol/L in female [13].

### Mediating metabolic indicators

Five groups of mediating metabolic indicators associated with MASLD and hyperuricemia were included in this study: (1) the lipid and glucose metabolism group included low-density lipoprotein cholesterol (LDL-C), total cholesterol, remnant cholesterol (RC), and triglyceride-glucose (TyG) index. The RC was derived by subtracting LDL-C and HDL-C from total cholesterol [14]. The TyG index, an established surrogate of IR, was calculated as the natural logarithm of [TG (mmol/L) × 88.57 × FBG (mmol/L) × 9] [15]; (2) hepatic function group, consisting of alanine aminotransferase (ALT), aspartate aminotransferase, gamma-glutamyl transferase (GGT), albumin (ALB), and alkaline phosphatase (ALP), and total bilirubin (TBIL); (3) renal function group, consisting of serum creatinine, blood urea nitrogen, and UA; and (4) inflammatory and immune response markers group, composed of white blood cell (WBC) count, lymphocyte percentage, neutrophil percentage, and the systemic immune-inflammation index (SII). The SII index is a composite marker reflecting systemic inflammation and immune status, calculated as platelet count × neutrophil count/lymphocyte count [16]; (5) The aging biomarker group was represented by PhenoAgeAccel, which was derived from a multivariable analysis of mortality risk. It is calculated using clinical parameters, including ALB, ALP, serum creatinine, FBG, lymphocyte percentage, mean cell volume, red blood cell distribution width, and WBC count [17].

### Statistical analysis

Continuous variables are expressed as mean ± standard deviation for normally distributed data or as median (interquartile range) for skewed distributions. Categorical variables are summarized as frequencies and percentages. The primary outcomes of this study were the incidence of MASLD and hyperuricemia, which were analyzed separately. Cox proportional hazards regression models were employed to estimate hazard ratios (HRs) and 95% confidence intervals (CIs) to assess the association between metabolic-obesity phenotypes and the risks of MASLD and hyperuricemia. Kaplan–Meier survival analyses were also performed to compare cumulative incidence across phenotypes, with group differences assessed using the log-rank test. Restricted cubic spline (RCS) models were used to assess the dose–response associations between baseline BMI and the risks of MASLD and hyperuricemia in metabolically healthy and unhealthy individuals. Knots were placed at the 5th, 35th, 65th, and 95th percentiles, with the estimated inflection point as the reference. An inflection-point analysis using a two-segment Cox model was further performed to interpret these nonlinear trends. Subgroup analyses were conducted to examine effect modification by baseline age ( < 40 years against ≥ 40 years) and sex (male against female).

Covariates were selected based on causal inference principles using directed acyclic graphs (DAGs) and study objectives (Figure S2). Age and sex were identified as confounders influencing both exposure and outcomes and included in the main Cox models. To avoid overadjustment, metabolic variables defining both exposure and outcomes were not re-adjusted, while liver and metabolic biomarkers were considered potential mediators and examined separately. Sensitivity analyses were performed to assess the robustness of the findings: (1) redefining obesity using the Chinese BMI classification (BMI ≥ 28.0 kg/m^2^) [18]; (2) applying the Adult Treatment Panel III criteria [19], which define metabolic health as having fewer than two abnormal metabolic components; (3) redefining the outcome as ultrasound-defined hepatic steatosis independent of metabolic components; and (4) fitting a discrete-time hazards model with a complementary log–log link to account for potential interval censoring.

We conducted a mediation analysis to evaluate whether and to what extent metabolic indicators beyond conventional definitions of metabolic health accounted for the associations between MHO, MASLD, and hyperuricemia risk. Following a two-stage regression approach [20], two models were constructed: a linear regression model for the metabolic indicators (mediators) and a Cox proportional hazards regression model for MASLD and hyperuricemia (outcomes). Based on this framework, the total association between MHO and disease risk was decomposed into a natural indirect effect, the proportion of the association explained by metabolic indicators, and the natural direct effect, the association independent of metabolic indicators [21]. To obtain robust 95% CIs, 5,000 bootstrapped samples were used to estimate the total, indirect, and direct effects. The mediation proportion was calculated as the ratio of the natural indirect effect to the total effect [22].

All statistical analyses were conducted using R software (version 4.4.2). For mediation analyses, the Benjamini–Hochberg procedure was applied to control type I error inflation from multiple comparisons, maintaining the false discovery rate at 0.05. All other analyses were two-sided, and a *p*-value < 0.05 was considered statistically significant.

We followed the STROBE reporting guideline and submitted the completed STROBE checklist as Supplementary Material.

## Results

### Characteristics of the study population

The baseline characteristics of participants across metabolic-obesity phenotypes are presented in Tables 1 and S2. Of the 17,040 individuals in the study, 11,712 were enrolled in the MASLD cohort, and 13,846 were part of the hyperuricemia cohort. The median age at baseline was 36.0 years (IQR, 30.0–42.0), with 47.9% and 51.2% of males in the two cohorts, respectively. At baseline, metabolically healthy individuals comprised 56.6% (*N* = 6,636) of the MASLD cohort and 47.8% (*N* = 6,621) of the hyperuricemia cohort, with 417 (6.3%) and 624 (9.4%) classified as MHO, respectively. Although MHO individuals had a more favorable metabolic profile than those with MUO, they still exhibited less favorable metabolic parameters and a higher PhenoAge compared with the MHNO group.

**Table 1:** Baseline characteristics of the study participants according to metabolic-obesity phenotypes in the MASLD cohort

Characteristics	Over all (n **=** 11,712)	MHNO (n = 6,219)	MHO (n = 417)	MUNO (n **=** 4,145)	MUO (n = 931)
Age (years), Mean ± SD	37.27 ± 9.70	35.72 ± 7.99	36.72 ± 9.62	39.06 ± 11.09	39.87 ± 11.48
PhenoAge (years), Mean ± SD	29.08 ± 10.27	27.20 ± 8.51	29.42 ± 10.12	30.98 ± 11.55	32.95 ± 12.25
Male, n (%)	5,610 (47.89)	2,509 (40.34)	261 (62.59)	2,139 (51.60)	701 (75.30)
BMI (kg/m^2^ ), Mean ± SD	22.16 ± 2.29	21.29 ± 1.63	26.26 ± 1.17	22.05 ± 1.68	26.65 ± 1.57
SBP (mmHg), Mean ± SD	118.96 ± 15.42	111.79 ± 10.07	116.74 ± 8.15	126.59 ± 16.50	133.90 ± 15.62
DBP (mmHg), Mean ± SD	71.77 ± 10.22	67.77 ± 7.57	70.29 ± 6.81	76.00 ± 10.93	80.32 ± 10.90
FBG (mmol/L), Median (IQR)	4.81 (4.50–5.13)	4.75 (4.46–5.02)	4.78 (4.48–5.06)	4.90 (4.56–5.29)	4.96 (4.61–5.38)
TC (mmol/L), Mean ± SD	4.86 ± 0.81	4.91 ± 0.89	4.98 ± 0.84	4.92 ± 1.00	5.08 ± 0.98
Triglyceride (mmol/L), Median (IQR)	0.97 (0.73–1.32)	0.85 (0.67–1.10)	1.02 (0.77–1.27)	1.14 (0.83–1.68)	1.43 (0.99–1.97)
HDL-C (mmol/L), Mean ± SD	1.43 ± 0.31	1.53 ± 0.28	1.39 ± 0.25	1.33 ± 0.30	1.23 ± 0.26
LDL-C (mmol/L), Mean ± SD	3.09 ± 0.71	3.03 ± 0.69	3.16 ± 0.66	3.12 ± 0.73	3.31 ± 0.77
Scr (μmol/L), Median (IQR)	64.80 (55.00–77.00)	62.00 (54.00–74.30)	72.00 (57.90–83.00)	66.00 (55.20–77.20)	74.00 (63.40–83.00)
Uric Acid (μmol/L), Mean ± SD	310.45 ± 78.46	296.10 ± 73.64	336.66 ± 77.72	318.19 ± 78.94	360.14 ± 79.85
ALT (U/L), Median (IQR)	16.00 (12.00–23.00)	15.00 (12.00–21.00)	20.00 (15.00–28.00)	17.00 (13.00–24.00)	22.00 (16.50–31.00)
AST (U/L), Median (IQR)	19.00 (17.00–23.00)	19.00 (16.00–22.00)	21.00 (17.00–25.00)	20.00 (17.00–24.00)	22.00 (18.00–26.00)
GGT (U/L), Median (IQR)	17.00 (13.00–25.00)	16.00 (12.00–22.00)	20.00 (15.00–27.00)	18.00 (14.00–27.00)	26.00 (18.00–38.00)
ALP (U/L), Median (IQR)	69.00 (57.00–84.00)	66.00 (55.00–81.00)	71.00 (61.00–85.00)	72.00 (60.00–87.00)	75.00 (62.00–88.00)
Albumin (g/L), Mean ± SD	46.05 ± 2.85	45.90 ± 2.71	45.85 ± 2.57	46.23 ± 3.03	46.32 ± 2.99
RBC count (×10^12^/L), Mean ± SD	4.84 ± 0.52	4.75 ± 0.49	4.97 ± 0.51	4.90 ± 0.52	5.11 ± 0.52
WBC count (×10^9^/L), Mean ± SD	5.89 ± 1.44	5.73 ± 1.39	6.12 ± 1.37	6.00 ± 1.47	6.38 ± 1.48
PLT count (×10^9^/L), Mean ± SD	239.56 ± 52.75	238.16 ± 51.91	243.68 ± 55.54	240.50 ± 53.77	242.96 ± 52.16
Lym Percentage (%), Mean ± SD	35.60 ± 7.55	35.99 ± 7.54	35.73 ± 7.27	35.15 ± 7.62	34.94 ± 7.34
SII, Median (IQR)	362.57 (274.09–480.00)	354.38 (269.53–467.27)	372.38 (282.00–465.76)	372.15 (279.00–490.71)	373.89 (285.31–500.53)

ALP, alkaline phosphatase; ALT, alanine aminotransferase; AST, aspartate aminotransferase; BMI, body mass index; DBP, diastolic blood pressure; FBG, fasting blood glucose; GGT, gamma-glutamyl transferase; HDL-C, high-density lipoprotein cholesterol; LDL-C, low-density lipoprotein cholesterol; Lym, lymphocyte; MHO, metabolically healthy obese; MHNO, metabolically healthy non-obese; MUO, metabolically unhealthy obese; MUNO, metabolically unhealthy non-obese; PLT, platelet; RBC, red blood cell; SBP, systolic blood pressure; Scr, serum creatinine; SII, systemic immune-inflammation index; TC, total cholesterol; WBC, white blood cell

Continuous variables are presented as mean ± standard deviation (SD) for normally distributed data or as median (interquartile range, IQR) for skewed distributions. Categorical variables are expressed as counts and percentages

Obesity was defined as BMI ≥25 kg/m^2^ according to the WHO Western Pacific Regional criteria

### Association between metabolic-obesity phenotypes and MASLD

Across 40,494 person-years of follow-up, 3,756 participants developed MASLD, yielding an incidence rate of 92.7 per 1,000 person-years. The associations between metabolic-obesity phenotypes and MASLD risk are illustrated in Table 2. After adjusting for age and sex, the HRs (95% CI) for MASLD, compared with the MHNO group, were 1.86 (1.73–2.01) for MUNO, 3.54 (3.09–4.05) for MHO, and 4.25 (3.85–4.69) for MUO. Individuals with MHO had a significantly increased risk of MASLD compared with MHNO, while the MUO group exhibited the highest overall risk. A significant trend was observed across phenotypes regardless of metabolic health status (*p* for trend < 0.001). Consistently, the Kaplan–Meier curves showed the lowest cumulative incidence in the MHNO group and the highest in the MUO group (Figure S3), with MHO participants exhibiting intermediate incidence between MHNO and MUO (*p* < 0.001, log-rank test).

**Table 2:** Associations between metabolic-obesity phenotypes and the risks of MASLD and hyperuricemia

Metabolic-obesity phenotypes	Cases	Preson-years	Incidence rate (cases per 1000 person-years)	Un-adjusted HR (95% CI）	Age- and Sex-adjusted HR (95% CI）
**MASLD**					
MHNO	1,274	23,146.1	55.0	Ref.	Ref.
MUNO	1,585	13,906.1	114.0	2.12 (1.97–2.29)	1.86 (1.73–2.01)
MHO	252	1,159.4	217.4	4.32 (3.77–4.94)	3.54 (3.09–4.05)
MUO	645	2,282.6	282.6	5.84 (5.31–6.43)	4.25 (3.85–4.69)
*p* for trend				*p* < 0.001	*p* < 0.001
**Hyperuricemia**					
MHNO	931	22,665.1	41.1	Ref.	Ref.
MUNO	1,231	17,273.0	71.3	1.75 (1.61–1.91)	1.57 (1.44–1.71)
MHO	211	2,096.3	100.7	2.49 (2.15–2.90)	1.92 (1.65–2.23)
MUO	921	7,131.2	129.2	3.24 (2.96–3.55)	2.36 (2.14–2.60)
*p* for trend				*p* < 0.001	*p* < 0.001

CI, confidence interval; HR, hazard ratio; MASLD, metabolic dysfunction-associated steatotic liver disease; MHO, metabolically healthy obese; MHNO, metabolically healthy non-obese; MUO, metabolically unhealthy obese; MUNO, metabolically unhealthy non-obese

Obesity was defined as BMI ≥25 kg/m^2^ according to the WHO Western Pacific Regional criteria

**Fig. 2:**
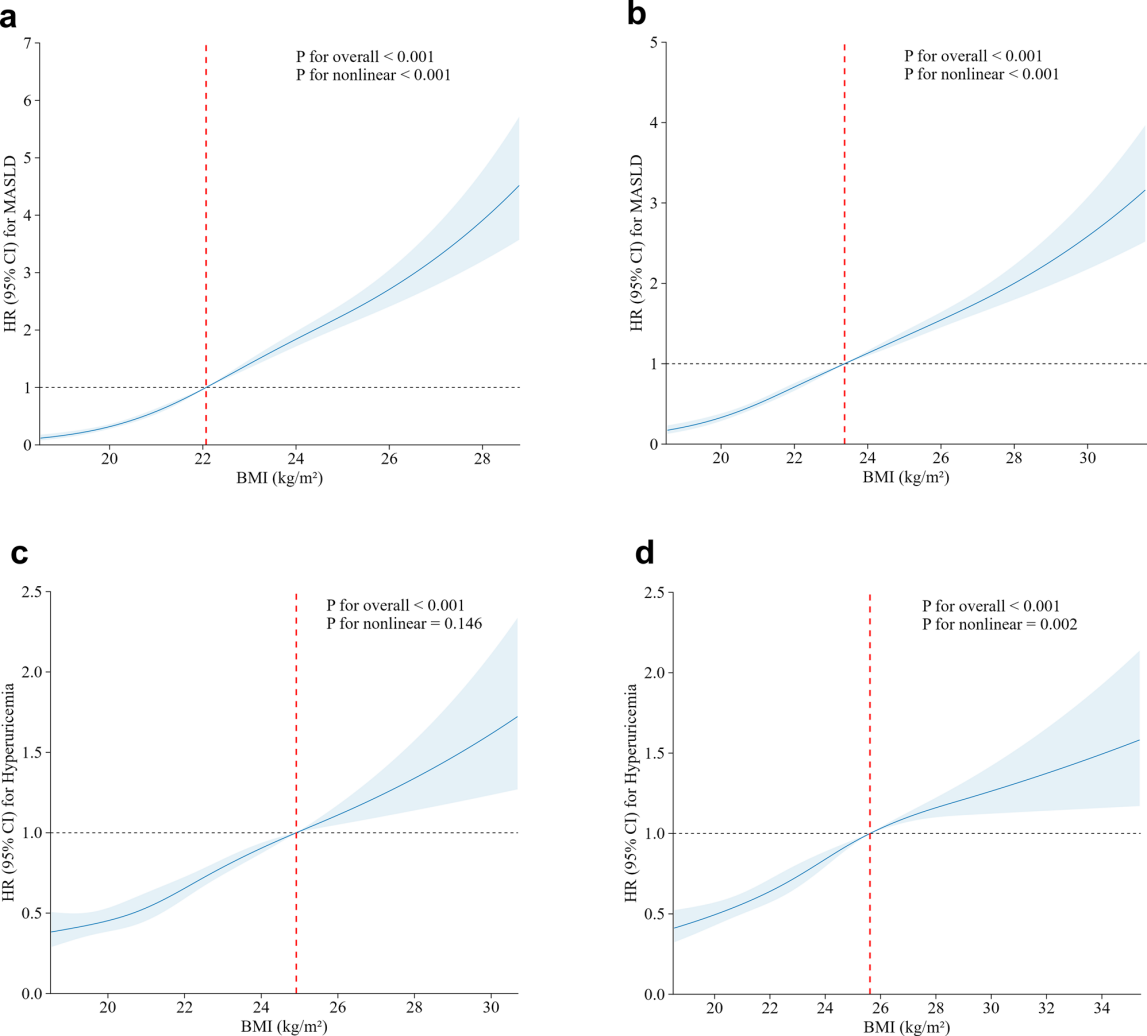
Dose-response relationship between BMI level and the risk of MASLD and hyperuricemia. The associations between BMI and the risk of incident MASLD in metabolically healthy (**a**) and unhealthy (**b**) individuals, and incident hyperuricemia in metabolically healthy (**c**) and unhealthy (**d**) individuals. Hazard ratios (HRs) and 95% confidence intervals (CIs) were estimated using restricted cubic spline Cox regression models, with the estimated inflection point as the reference. All models were adjusted for age and sex. Solid lines represent HRs, and shaded areas indicate 95% CIs. BMI, body mass index; MASLD, metabolic dysfunction-associated steatotic liver disease

As shown in Fig. 2a–b, baseline BMI exhibited a significant nonlinear association with MASLD risk in both metabolically healthy and unhealthy groups (all *p* for nonlinearity < 0.001). The inflection-point analysis identified BMI thresholds of 22.07 kg/m^2^ and 23.37 kg/m^2^, respectively.The subgroup analyses based on sex and age are presented in Table 3. The relationship between MHO and MASLD was significant for both sexes (*p* < 0.001), with a more pronounced effect in females (HR = 5.81, 95% CI: 4.57–7.37) than in males (HR = 2.77, 95% CI: 2.35–3.26). In the age-stratified analysis, individuals with MHO aged < 40 years had a slightly higher risk (HR = 3.62, 95% CI: 3.08–4.24) than those aged ≥ 40 years (HR = 3.30, 95% CI: 2.54–4.30). In sensitivity analyses (Tables S3, S4, and S5), using alternative definitions and modeling approaches did not substantially alter the associations between metabolic-obesity phenotypes and MASLD.

**Table 3:** Subgroup analyses of the associations between metabolic-obesity phenotypes and the risks of MASLD and hyperuricemia

Metabolic-obesity phenotypes	Male HR (95% CI）^a^	Female HR (95% CI）^a^	Age < 40 years HR (95% CI）^b^	Age ≥ 40 years HR (95% CI）^b^
**MASLD**				
MHNO	Ref.	Ref.	Ref.	Ref.
MUNO	1.64 (1.50–1.80)	2.27 (2.00–2.58)	1.77 (1.62–1.94)	2.03 (1.78–2.32)
MHO	2.77 (2.35–3.26)	5.81 (4.57–7.37)	3.62 (3.08–4.24)	3.30 (2.54–4.30)
MUO	3.70 (3.30–4.14)	6.08 (4.98–7.43)	4.72 (4.19–5.32)	3.85 (3.25–4.56)
*p* for trend	*p* < 0.001	*p* < 0.001	*p* < 0.001	*p* < 0.001
**Hyperuricemia**				
MHNO	Ref.	Ref.	Ref.	Ref.
MUNO	1.38 (1.25–1.54)	1.86 (1.59–2.18)	1.36 (1.23–1.51)	1.93 (1.64–2.27)
MHO	1.63 (1.38–1.93)	2.83 (2.05–3.91)	1.81 (1.52–2.16)	2.10 (1.56–2.81)
MUO	2.04 (1.83–2.26)	3.91 (3.19–4.79)	2.23 (2.00–2.50)	2.56 (2.14–3.06)
*p* for trend	*p* < 0.001	*p* < 0.001	*p* < 0.001	*p* < 0.001

CI, confidence interval; HR, hazard ratio; MASLD, metabolic dysfunction-associated steatotic liver disease; MHNO, metabolically healthy non-obese; MHO, metabolically healthy obese; MUNO, metabolically unhealthy non-obese; MUO, metabolically unhealthy obese

^a^Model was adjusted for age; ^b^Model was adjusted for sex

Obesity was defined as BMI ≥25 kg/m^2^ according to the WHO Western Pacific Regional criteria

### Association between metabolic-obesity phenotypes and hyperuricemia

Over a follow-up period of 49,165 person-years, 3,294 participants were diagnosed with hyperuricemia, yielding an incidence rate of 67 per 1,000 person-years. As presented in Table 2, individuals in MUNO, MHO, and MUO groups had a higher risk of hyperuricemia compared with the MHNO group, with HRs (95% CI) of 1.57 (1.44–1.71), 1.92 (1.65–2.23), and 2.36 (2.14–2.60), respectively. The Kaplan–Meier curves further demonstrated a stepwise increase in cumulative incidence across phenotypes (Figure S3), with MHO participants showing intermediate incidence between MHNO and MUO groups (*p* < 0.001, log-rank test).

Figure 2c–d shows the association between baseline BMI and hyperuricemia risk. The relationship was approximately linear among metabolically healthy participants (*p* for nonlinearity = 0.146) but nonlinear among metabolically unhealthy individuals (*p* = 0.002). The inflection-point analysis identified BMI thresholds of 24.92 kg/m^2^ and 25.62 kg/m^2^, respectively. The subgroup analyses stratified according to sex and age are presented in Table 3. Females with MHO exhibited a higher risk of hyperuricemia (HR = 2.83, 95% CI: 2.05–3.91) than their male counterparts (HR = 1.63, 95% CI: 1.38–1.93). The association of MHO with hyperuricemia was more evident in those aged ≥ 40 years (HR = 2.10, 95% CI: 1.56–2.81) than in younger individuals (HR = 1.81, 95% CI: 1.52–2.16), indicating a higher vulnerability in the older population. The associations between metabolic-obesity phenotypes and hyperuricemia remained consistent in the sensitivity analyses (Tables S3 and S5).

### Mediation analysis of metabolic indicators on the association between MHO and MASLD

**Fig. 3:**
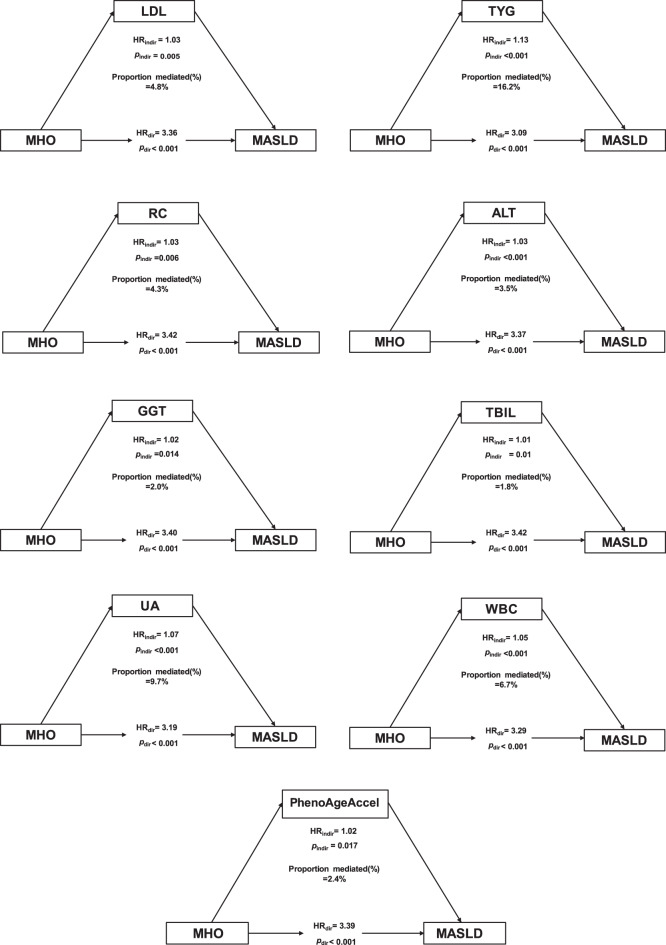
Mediation analysis of metabolic indicators on the associations between MHO and the risks of MASLD. MASLD, metabolic dysfunction-associated steatotic liver disease; MHO, metabolically healthy obese; HR, hazard ratio

Table S6 presents the results of the mediation analysis of metabolic indicators in the association between MHO and MASLD. After adjusting for sex and age, nine metabolic indicators were identified as mediators of this association to varying degrees (Fig. 3). Among them, the TyG index and UA explained the largest proportions of the total association, 16.2% and 9.7%, respectively. In contrast, the effects of LDL-C, RC, ALT, GGT, TBIL, and WBC were relatively modest. PhenoAgeAccel also showed an independent and notable indirect effect, suggesting that biological aging may partly explain the observed association with MASLD. Overall, metabolic indicators related to lipid and glucose metabolism contributed more to the mediation pathway than those from other metabolic groups.

### Mediation analysis of metabolic indicators on the association between MHO and hyperuricemia

Table S7 presents the results of the mediation analysis of metabolic indicators in the association between MHO and hyperuricemia. After adjusting for sex and age, four metabolic indicators were identified as mediators of this association to varying extents (Figure S1). Among them, the TyG index showed the largest explained proportion, accounting for 13.8%. In contrast, the contributions of GGT, ALB, and WBC levels were relatively modest. Overall, the lipid and glucose metabolism group remained the predominant contributor to the mediation pathway linking MHO to hyperuricemia, playing a more substantial role than other metabolic groups.

## Discussion

This cohort study evaluated MASLD and hyperuricemia risk across different metabolic-obesity phenotypes. Compared with MHNO individuals, those with MHO exhibited significantly increased risks for both outcomes. These findings suggest that obesity may be an independent pathogenic factor. Mediation analysis identified several metabolic indicators statistically associated with these associations under the modeling assumptions. These findings suggest that individuals with MHO, although considered metabolically healthy, may still have residual metabolic disturbances, with their increased disease risk potentially reflecting both direct influences of obesity and indirect pathways involving metabolic factors.

Our findings are consistent with previous research demonstrating that MHO is not a benign phenotype. Yoosoo Chang et al. previously reported a significant association between MHO and nonalcoholic fatty liver disease [23], and similar evidence from Chinese populations has shown an increased risk of hyperuricemia among individuals with MHO [24]. By applying the updated MASLD definition [25] and conducting parallel analyses for both MASLD and hyperuricemia within a unified metabolic framework, our study extends previous evidence and provides a more comprehensive understanding of the long-term implications of this phenotype. We also observed stronger associations in females, consistent with prior findings that obesity-related alterations in estrogen signaling may promote hepatic lipid accumulation and reduce renal uric acid excretion, thereby increasing disease susceptibility [26, 27]. In summary, our results indicate that even obese individuals classified as metabolically healthy under traditional criteria remain at elevated risk for MASLD and hyperuricemia, underscoring the importance of strengthened metabolic assessment and weight management for early prevention. Building on these findings, we conducted mediation analyses to further explore the metabolic pathways potentially explaining these associations, providing clinical evidence that refines current understanding of how MHO relates to metabolic diseases.

Previous studies have illustrated that lipid parameters may mediate the relationship between BMI and metabolic diseases [28, 29]. Several studies indicated that TG and other lipid markers partially explained the association between BMI and MASLD [30, 31]. Similarly, HDL-C and TG levels are associated with BMI and gout [32]. Consistently, our mediation analysis illustrated that the associations between MHO and MASLD and hyperuricemia risk were partially explained by a range of metabolic disturbances.

In the MASLD analysis, the IR marker TyG explained 16.2% of the total effect, suggesting that obesity may promote hepatic lipid accumulation and lipotoxicity through IR [33], even with normal FBG and TG levels. The indirect effects of LDL-C and RC indicate that impaired cholesterol clearance may contribute to intrahepatic fat accumulation through enhanced hepatic lipid uptake and reduced cholesterol efflux [34]. UA accounted for 9.7% of the explained proportion, implying a possible shared pathogenic pathway between uric acid metabolism and hepatic steatosis [35, 36]. Inflammatory indicators (WBC) and liver function markers (ALT, GGT, and TBIL) also showed modest indirect effects, highlighting the roles of chronic inflammation and hepatocellular injury in the development of MASLD. Notably, TBIL, an endogenous antioxidant, may reflect hepatic dysfunction and redox imbalance associated with oxidative stress, suggesting that sustained oxidative burden constitutes a potential mechanistic link between metabolic dysfunction and disease progression [37, 38]. Aging is an irreversible, systemic biological process characterized by a progressive decline in various physiological functions. PhenoAge is considered an effective tool for evaluating the degree of biological aging in individuals. Our analysis identified a distinct indirect effect of PhenoAgeAccel with the relationship between MHO and MASLD, suggesting that MHO may reflect accelerated biological aging. This aging process may promote metabolic dysfunction through mitochondrial impairment, oxidative stress, and epigenetic dysregulation [17, 39]. PhenoAge provides a novel age-related perspective for assessing MASLD in obese individuals, contributing to a more comprehensive evaluation of metabolic health.

In hyperuricemia analysis, the TyG index explained 13.8% of the total effect, highlighting the central role of IR in uric acid metabolism. Latent IR may contribute to hyperuricemia in individuals with MHO by upregulating urate transporter 1 and glucose transporter 9 and promoting uric acid precursor accumulation through impaired glycolytic pathways [40–42]. The indirect effects of GGT and ALB suggest that hepatic impairment may indirectly increase UA levels by altering purine metabolism or reducing the hepatic clearance of UA precursors [43]. Furthermore, the observed WBC role suggests that chronic inflammation may contribute to impaired UA homeostasis [44]. Together with the MASLD findings, these results indicate that both conditions share convergent metabolic pathways driven by IR, dyslipidemia, and oxidative stress. Specifically, IR perturbs lipid and purine metabolism, whereas elevated oxidative stress further stimulates hepatic uric acid synthesis and exacerbates hepatocellular injury. In parallel, obesity-associated adipokine dysregulation amplifies inflammatory and oxidative cascades, thereby perpetuating metabolic dysfunction [42, 45]. This integrative framework underscores a shared pathogenic basis and provides a mechanistic rationale for the frequent coexistence of these conditions in individuals with MHO. It also refines the current understanding of metabolic health by revealing the inherent heterogeneity within this phenotype.

Although this study included a large dataset on metabolic-obesity phenotypes and explored mediating metabolic indicators linking obesity to MASLD and hyperuricemia through mediation analysis, several limitations should be addressed. First, MASLD was diagnosed by abdominal ultrasonography rather than liver biopsy, the current gold standard. However, ultrasonography is widely used in epidemiological studies and has proven reliable for detecting hepatic steatosis [46]. Second, due to the inherent limitations of the health-examination cohort, detailed information on lifestyle factors and medication use was unavailable, which may have led to residual confounding. Future studies incorporating more comprehensive data on potential confounders are warranted to further validate these findings. Third, combining MetS criteria with HOMA-IR could have improved the accuracy of metabolic health classification, but this was not feasible because fasting insulin data were unavailable for many participants. Fourth, metabolic health is dynamic and may change over time, and unobserved transitions could lead to mild exposure misclassification and affect causal inference. Future studies incorporating repeated measurements or state-transition models will help further validate these temporal relationships. Finally, the study population mainly consisted of relatively young Chinese adults undergoing routine health examinations. Therefore, the generalizability of these findings to older or other populations may be limited and requires further validation.

## Conclusion

MHO increases the risks of MASLD and hyperuricemia even with normal levels of conventional metabolic indicators, indicating that current definitions may not fully reflect obesity-related metabolic risks. Mediation analysis revealed latent metabolic disturbances in individuals with MHO, particularly those involving IR, chronic inflammation, and biological aging. These findings highlight the importance of supplementing conventional metabolic assessments with mediating metabolic indicators in obese populations to improve early risk identification and inform more effective prevention strategies.

## Electronic supplementary material

Below is the link to the electronic supplementary material.

Supplementary Material 1

## Data Availability

The original data supporting the study are available from the first author or corresponding author subject to compliance with laws and regulations.

## Abbreviations

ALB: Albumin
ALP: Alkaline phosphatase
ALT: Alanine aminotransferase
BMI: Body mass index
CI: Confidence interval
FBG: Fasting blood glucose
GGT: Gamma-glutamyl transferase
HDL-C: High-density lipoprotein cholesterol
HR: Hazard ratio
IR: Insulin resistance
LDL-C: Low-density lipoprotein cholesterol
MetS: Metabolic syndrome
MHO: Metabolically healthy obese
MHNO: Metabolically healthy non-obese
MUO: Metabolically unhealthy obese
MUNO: Metabolically unhealthy non-obese
RC: Remnant cholesterol
SII: Systemic immune-inflammation index
TBIL: Total bilirubin
TG: Triglyceride
TyG: Triglyceride-glucose
UA: Uric acid
WBC: White blood cell
