# Supplementary material for: Metabolically healthy obesity increases the risks of MASLD and hyperuricemia: a cohort study with mediation analysis
Source: BMC Endocr Disord. 2025 Nov 27;26:3. doi: 10.1186/s12902-025-02106-9 (PMC12763833; doi:10.1186/s12902-025-02106-9)
Supplement: Supplementary file 1 — Supplementary Material 1 [file 12902_2025_2106_MOESM1_ESM.docx]

***Supplementary Figures (1-3) and Tables (1–7) for***

**Metabolically Healthy Obesity Increases the Risks of MASLD and Hyperuricemia: A Cohort Study with Mediation Analysis**

**Authors: Yiyun Liu^1,2^, Tianchen Qian^1,3^, Yiyang Zheng^1^, Aiming Liu^2^, Lei Xu^1,3*^, Guoliang Ye^1*^, Jiarong Xie^1,3*^**

**Authors’ affiliations:**

**^1^ Department of Gastroenterology, The First Affiliated Hospital of Ningbo University,**

**Ningbo, 315010, China.**

**^2^ Health Science Center, Ningbo University, Ningbo, 315211, Zhejiang, China.**

^3^ Department of Gastroenterology, The First Affiliated Hospital, College of Medicine, Zhejiang University. Hangzhou, 310003, China.

***Corresponding authors:**

Dr. Jiarong Xie, Department of Gastroenterology, The First Affiliated Hospital of Ningbo University, Ningbo, China. No.59. Liuting Street, Haishu District, Ningbo 315000, China.

E-mail: cheryl.xiejiarong@gmail.com

Prof. Guoliang Ye, Department of Gastroenterology, The First Affiliated Hospital of Ningbo University, Ningbo, China. No.59. Liuting Street, Haishu District, Ningbo 315000, China.

E-mail: yeguoliang@nbu.edu.cn

Dr. Lei Xu, Department of Gastroenterology, The First Affiliated Hospital of Ningbo University, Ningbo, China. No.59. Liuting Street, Haishu District, Ningbo 315000, China.

Phone: 0086-13486659126; Fax: 0086-574-87291583; E-mail: xulei22@163.com

**Supplement :**

**Supplementary Figure S1.** Mediation analysis of metabolic indicators on the association between MHO and the risk of hyperuricemia.

**Supplementary Figure S2.** Directed acyclic graphs of the hypothesized causal structures between MHO and MASLD and hyperuricemia.

**Supplementary Figure S3.** Kaplan**-**Meier survival curves for the incidence of MASLD and hyperuricemia according to metabolic**-**obesity phenotypes.

**Supplementary Table S1.** Summary of missing values for study variables.

**Supplementary Table S2.** Baseline characteristics of the study participants according to metabolic-obesity phenotypes in the hyperuricemia cohort.

**Supplementary Table S3.** Sensitivity analyses of the associations between metabolic**-**obesity phenotypes and the risks of MASLD and hyperuricemia, based on alternative definitions of obesity and metabolic health.

**Supplementary Table S4.** Sensitivity analyses of the associations between metabolic-obesity phenotypes and the risk of ultrasound-defined hepatic steatosis independent of metabolic components.

**Supplementary Table S5.** Sensitivity analyses of the associations between metabolic**-**obesity phenotypes and the risks of MASLD and hyperuricemia using a discrete-time hazards model.

**Supplementary Table S6.** Mediation analysis of metabolic indicators on the association between MHO and MASLD.

**Supplementary Table S7.** Mediation analysis of metabolic indicators on the association between MHO and hyperuricemia.


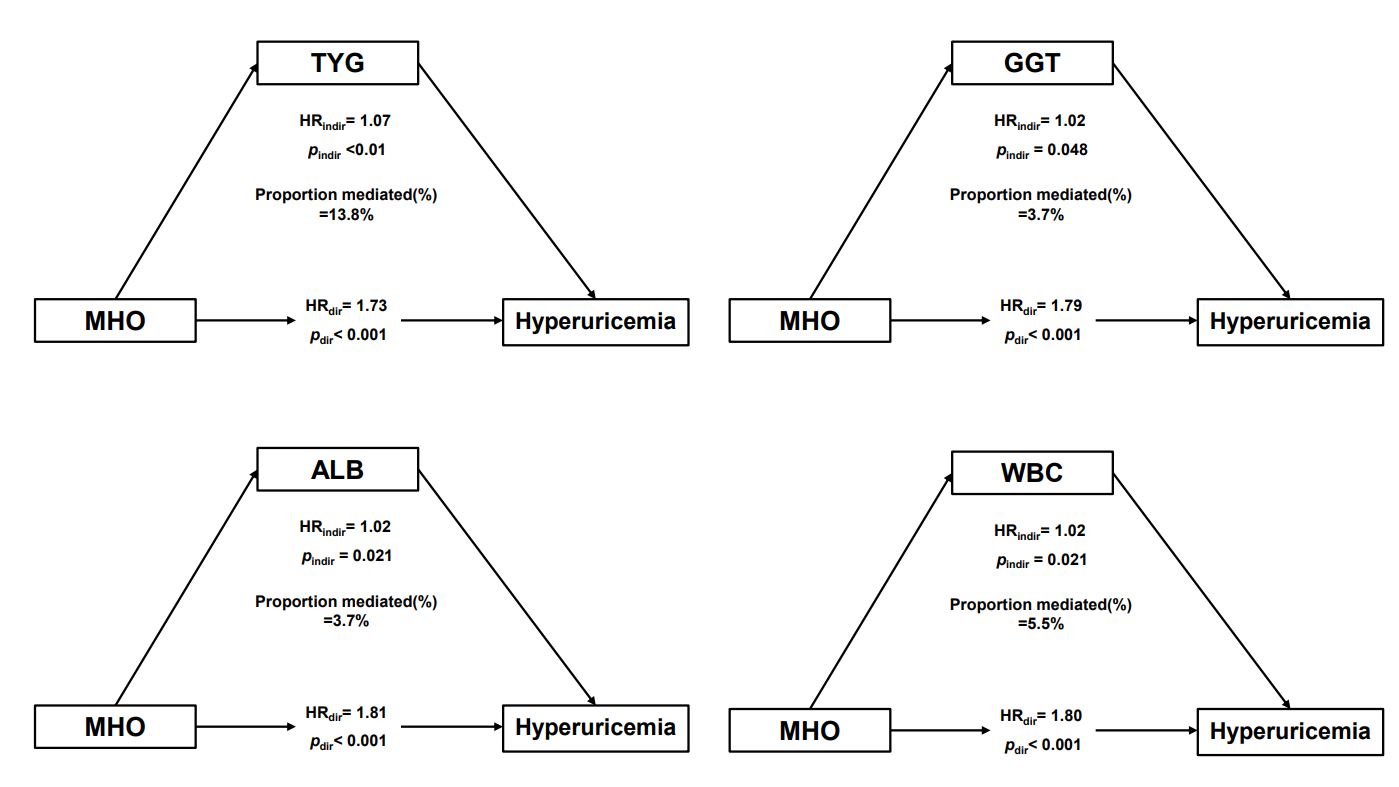


**Supplementary Figure S1.** Mediation analysis of metabolic indicators on the association between MHO and the risk of hyperuricemia. MHO, metabolically healthy obese; HR, hazard ratio.


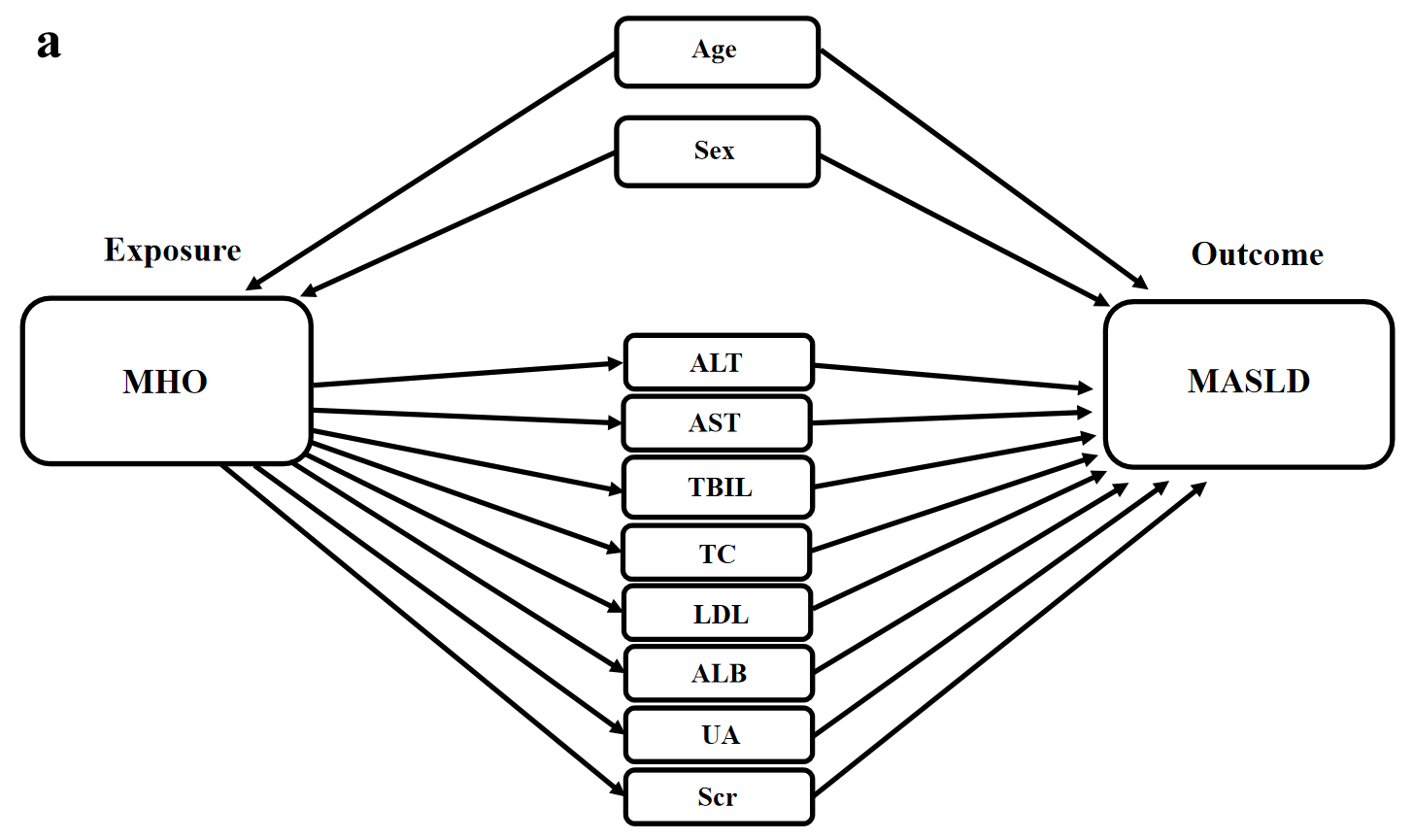


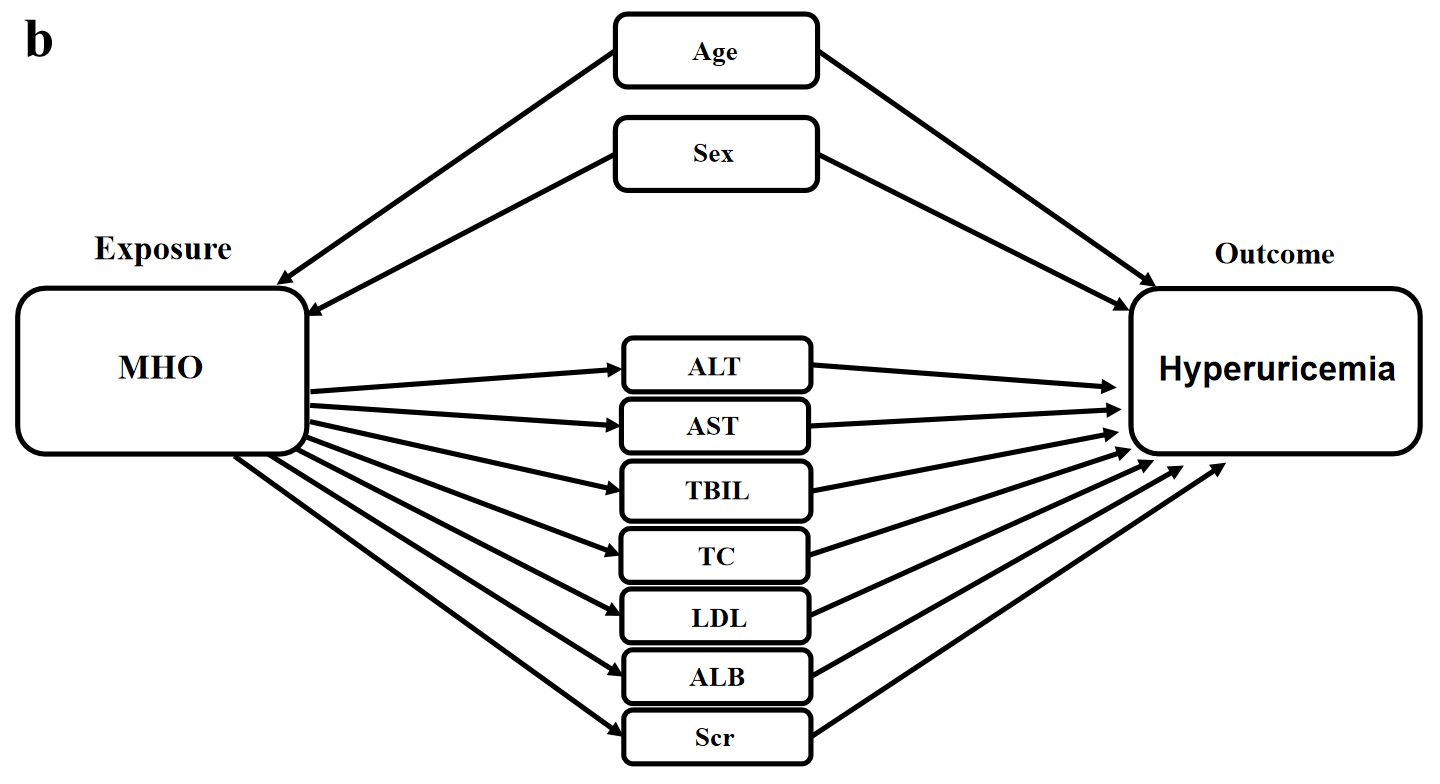


**Supplementary Figure S2.** Directed acyclic graphs of the hypothesized causal structures between MHO and MASLD and hyperuricemia.

ALB, albumin; ALT, alanine aminotransferase; AST, aspartate aminotransferase; LDL-C, low-density lipoprotein cholesterol; MASLD, metabolic dysfunction-associated steatotic liver disease; MHO, metabolically healthy obese; Scr, serum creatinine; TBIL, total bilirubin; TC, total cholesterol; UA, uric acid.


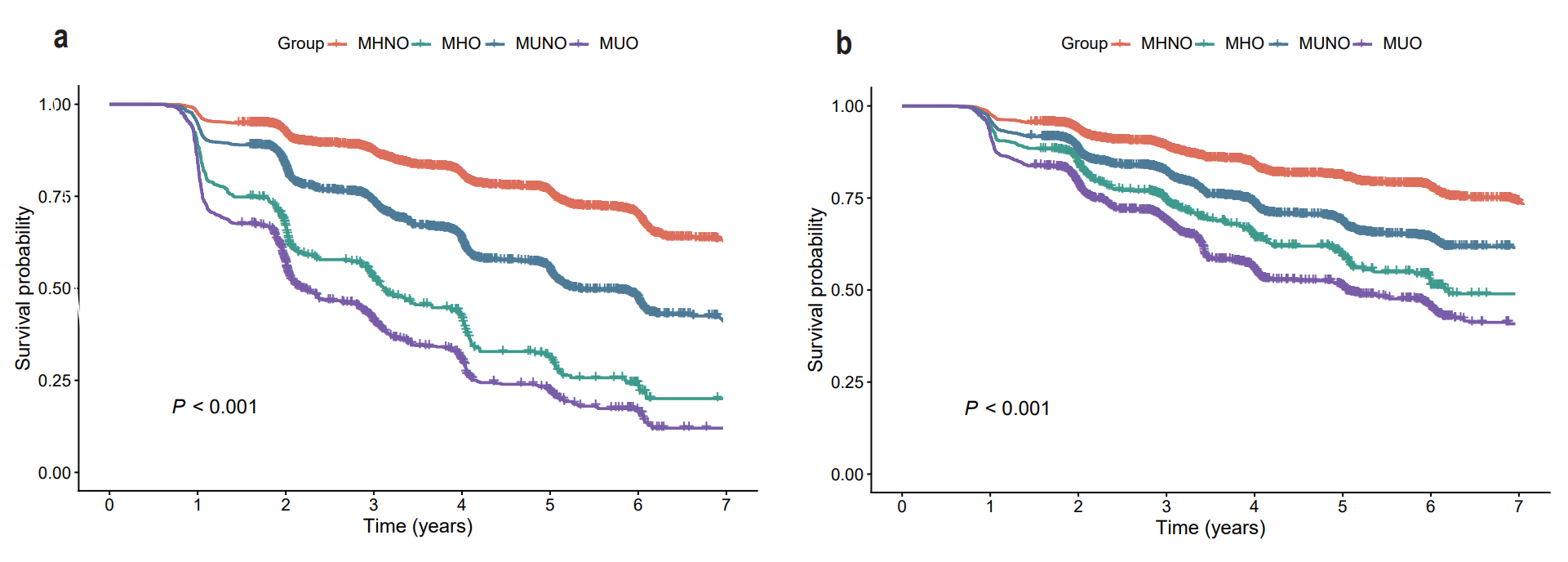


**Supplementary Figure S3.** Kaplan**-**Meier survival curves for the incidence of MASLD and hyperuricemia according to metabolic**-**obesity phenotypes.

Kaplan**-**Meier curves showing the cumulative incidence of (a) MASLD and (b) hyperuricemia across metabolic**-**obesity phenotypes. Group differences were tested using the log-rank test (*p* < 0.001). MASLD, metabolic dysfunction-associated steatotic liver disease; MHNO, metabolically healthy non-obese; MHO, metabolically healthy obese; MUO, metabolically unhealthy obese; MUNO, metabolically unhealthy non-obese.

**Supplementary Table S1.** Summary of missing values for study variables.

| **.Variable** | **Missing, n** | **Missing, %** |
| --- | --- | --- |
| Age | 0 | 0 |
| Sex | 0 | 0 |
| BMI | 0 | 0 |
| SBP | 35 | 0.19% |
| DBP | 35 | 0.19% |
| FBG | 10 | 0.05% |
| TC | 59 | 0.32% |
| Triglyceride | 59 | 0.32% |
| HDL-C | 59 | 0.32% |
| LDL-C | 59 | 0.32% |
| Scr | 0 | 0 |
| Uric Acid | 0 | 0 |
| ALT | 2 | 0.01% |
| AST | 2 | 0.01% |
| GGT | 2 | 0.01% |
| TBIL | 2 | 0.01% |
| ALP | 2 | 0.01% |
| Albumin | 2 | 0.01% |
| RBC count | 15 | 0.08% |
| WBC count | 15 | 0.08% |
| PLT count | 18 | 0.09% |
| Lym Percentage | 2 | 0.01% |

ALP, alkaline phosphatase; ALT, alanine aminotransferase; AST, aspartate aminotransferase; BMI, body mass index; DBP, diastolic blood pressure; FBG, fasting blood glucose; GGT, gamma-glutamyl transferase; HDL-C, high-density lipoprotein cholesterol; LDL-C, low-density lipoprotein cholesterol; Lym, lymphocyte; PLT, platelet; RBC, red blood cell; SBP, systolic blood pressure; Scr, serum creatinine; TBIL, total bilirubin; TC, total cholesterol; WBC, white blood cell.

**Supplementary Table S2.** Baseline characteristics of the study participants according to metabolic-obesity phenotypes in the hyperuricemia cohort.

| **Characteristics** | **1 Over all**  (n **=** 13,846) | **MHNO**  (n = 5,997) | **MHO**  (n = 624) | **1MUNO**  (n **=** 4,921) | **MUO**  (n = 2,304) |
| --- | --- | --- | --- | --- | --- |
| Age (years), Mean ± SD | 38.32 ± 9.97 | 35.93 ± 7.95 | 37.56 ± 8.90 | 40.23 ± 11.27 | 40.67 ± 10.60 |
| PhenoAge (years), Mean ± SD | 28.58 ± 8.74 | 27.20 ± 8.26 | 28.65 ± 7.24 | 30.02 ± 9.90 | 31.07 ± 6.95 |
| Male, n (%) | 7,094 (51.21) | 2,329 (38.84) | 424 (67.95) | 2,567 (52.16) | 1,774 (77.00) |
| BMI (kg/m^2^ ), Mean ± SD | 22.89 ± 2.81 | 21.34 ± 1.68 | 26.48 ± 1.41 | 22.28 ± 1.68 | 27.26 ± 2.01 |
| SBP (mmHg), Mean ± SD | 121.37 ± 16.35 | 111.76 ± 10.07 | 117.33 ± 8.08 | 127.30 ± 16.43 | 134.80 ± 15.79 |
| DBP (mmHg), Mean ± SD | 73.36 ± 10.93 | 67.77 ± 7.56 | 70.86 ± 7.03 | 76.48 ± 10.93 | 81.90 ± 11.13 |
| FBG (mmol/L), Median (IQR) | 4.86 (4.54 – 5.21) | 4.76 (4.47 – 5.02) | 4.79 (4.50 – 5.11) | 4.95 (4.61 – 5.38) | 5.10 (4.71 – 5.58) |
| TC (mmol/L), Mean ± SD | 5.00 ± 0.96 | 4.92 ± 0.89 | 5.04 ± 0.88 | 4.99 ± 1.01 | 5.22 ± 1.00 |
| Triglyceride (mmol/L), Median (IQR) | 1.06 (0.77 – 1.50) | 0.85 (0.67–1.10) | 1.08 (0.83 – 1.36) | 1.22 (0.88 – 1.78) | 1.66 (1.14 – 2.22) |
| HDL-C (mmol/L), Mean ± SD | 1.39 ± 0.31 | 1.54 ± 0.28 | 1.37 ± 0.25 | 1.32 ± 0.30 | 1.32 ± 0.30 |
| LDL-C (mmol/L), Mean ± SD | 3.15 ± 0.73 | 3.02 ± 0.69 | 3.22 ± 0.69 | 3.18 ± 0.75 | 3.40 ± 0.74 |
| Scr (μmol/L), Median (IQR) | 65.00 (55.00 – 76.00) | 61.00 (53.70 – 73.00) | 71.30 (60.00 – 80.00) | 65.00 (55.00 – 76.00) | 72.00 (62.00 – 79.50) |
| Uric Acid (μmol/L), Mean ± SD | 303.20 ± 62.25 | 285.04 ± 60.35 | 325.61 ± 55.69 | 306.03 ± 60.88 | 338.40 ± 52.95 |
| ALT (U/L), Median (IQR) | 18.00 (13.00 – 26.00) | 15.00 (11.00 – 21.00) | 22.00 (16.00 – 31.00) | 18.00 (13.00 – 26.00) | 27.00 (19.00 – 41.00) |
| AST (U/L), Median (IQR) | 20.00 (17.00 – 24.00) | 19.00 (16.00 – 22.00) | 21.00 (18.00 – 25.00) | 20.00 (17.00 – 24.00) | 23.00 (19.00 – 28.00) |
| GGT (U/L), Median (IQR) | 19.00 (14.00 – 29.00) | 16.00 (12.00 – 22.00) | 21.50 (16.00 – 32.00) | 20.00 (14.00 – 30.00) | 31.00 (21.00 – 46.00) |
| ALP (U/L), Mean ± SD | 73.88 ± 21.85 | 69.19 ± 20.57 | 76.16 ± 20.64 | 76.17 ± 21.92 | 80.59 ± 22.60 |
| Albumin (g/L), Mean ± SD | 46.12 ± 2.88 | 45.85 ± 2.70 | 45.88 ± 2.62 | 46.26 ± 3.02 | 46.58 ± 3.04 |
| RBC count (×10^12^/L), Mean ± SD | 4.89 ± 0.52 | 4.74 ± 0.49 | 5.04 ± 0.52 | 4.91 ± 0.51 | 5.16 ± 0.49 |
| WBC count (×10^9^/L), Mean ± SD | 6.02 ± 1.49 | 5.71 ± 1.38 | 6.15 ± 1.35 | 6.08 ± 1.50 | 6.62 ± 1.58 |
| PLT count (×10^9^/L), Mean ± SD | 242.20 ± 53.63 | 238.63 ± 52.31 | 245.29 ± 54.20 | 243.04 ± 53.74 | 248.87 ± 55.85 |
| Lym Percentage (%), Mean ± SD | 35.44 ± 7.48 | 35.97 ± 7.53 | 35.78 ± 6.98 | 35.03 ± 7.52 | 34.88 ± 7.33 |
| SII, Median (IQR) | 369.44 (279.02 – 486.43) | 356.40 (269.79 – 469.33) | 371.34 (281.87 – 462.00) | 378.00 (286.76 – 498.00) | 388.07 (291.92 – 513.21) |

ALP, alkaline phosphatase; ALT, alanine aminotransferase; AST, aspartate aminotransferase; BMI, body mass index; DBP, diastolic blood pressure; FBG, fasting blood glucose; GGT, gamma-glutamyl transferase; HDL-C, high-density lipoprotein cholesterol; LDL-C, low-density lipoprotein cholesterol; Lym, lymphocyte; MHNO, metabolically healthy non-obese; MHO, metabolically healthy obese; MUO, metabolically unhealthy obese; MUNO, metabolically unhealthy non-obese; PLT, platelet; RBC, red blood cell; SBP, systolic blood pressure; Scr, serum creatinine; SII, systemic immune-inflammation index; TC, total cholesterol; WBC, white blood cell.

Continuous variables are presented as mean ± standard deviation (SD) for normally distributed data or as median (interquartile range, IQR) for skewed distributions. Categorical variables are expressed as counts and percentages.

Obesity was defined as BMI ≥25 kg/m² according to the WHO Western Pacific Regional criteria.

**Supplementary Table S3.** Sensitivity analyses of the associations between metabolic-obesity phenotypes and

the risks of MASLD and hyperuricemia, based on alternative definitions of obesity and metabolic health.

| **Metabolic-obesity phenotypes** |  |  | **Based on the obesity classification criterion of BMI ≥ 28 kg/m²**  **HR (95% CI）^a^** |  |  | **Based on ATP III, metabolic health was defined as < 2 metabolic abnormalities.**  **HR (95% CI）^a^** |
| --- | --- | --- | --- | --- | --- | --- |
| **MASLD** |  |  |  |  |  |  |
| MHNO |  |  | Ref. |  |  | Ref. |
| MUNO |  |  | 1.88 (1.76 – 2.01) |  |  | 1.88 (1.72 – 2.05) |
| MHO |  |  | 4.68 (3.13 – 7.01) |  |  | 3.22 (2.92 – 3.54) |
| MUO |  |  | 5.31 (4.40 – 6.41) |  |  | 3.62 (3.22 – 4.06) |
| *p* for trend |  |  | *p* <0.001 |  |  | *p* <0.001 |
| **Hyperuricemia** |  |  |  |  |  |  |
| MHNO |  |  | Ref. |  |  | Ref. |
| MUNO |  |  | 1.58 (1.47 – 1.71) |  |  | 1.62 (1.47 – 1.78) |
| MHO |  |  | 1.97 (1.37 – 2.81) |  |  | 1.86 (1.68 – 2.05) |
| MUO |  |  | 2.49 (2.17 – 2.84) |  |  | 2.16 (1.95 – 2.38) |
| *p* for trend  ATP III, Adult Treatment Panel III; CI, confidence interval; HR, hazard ratio; MASLD, metabolic dysfunction -associated steatotic liver disease; MHNO, metabolically healthy non-obese; MHO, metabolically healthy obese; MUNO, metabolically unhealthy non-obese; MUO, metabolically unhealthy obese. ^a^Model was adjusted for age and sex.  Obesity was defined as BMI ≥25 kg/m² according to the WHO Western Pacific Regional criteria. |  |  | *p* <0.001 |  |  | *p* <0.001 |

**Supplementary Table S4.** Sensitivity analyses of the associations between

metabolic-obesity phenotypes and the risk of ultrasound-defined hepatic

steatosis independent of metabolic components.

| **Metabolic-obesity phenotypes** |  | **Age- and sex- adjusted**  **HR (95% CI）** |
| --- | --- | --- |
| MHNO |  | Ref. |
| MUNO |  | 1.75 (1.63 – 1.88) |
| MHO |  | 3.16 (2.76 – 3.62) |
| MUO  CI, confidence interval; HR, hazard ratio; MHNO, metabolically healthy non-obese; MHO, metabolically healthy obese; MUNO, metabolically unhealthy non-obese; MUO, metabolically unhealthy obese.  Obesity was defined as BMI ≥25 kg/m² according to the WHO Western Pacific Regional criteria. |  | 3.78 (3.43 – 4.17) |

**Supplementary Table S5.** Sensitivity analyses of the associations between

metabolic**-**obesity phenotypes and the risks of MASLD and hyperuricemia

using a discrete-time hazards model.

| **Metabolic-obesity phenotypes** |  |  | **Age- and sex- adjusted**  **HR (95% CI）** |
| --- | --- | --- | --- |
| **MASLD** |  |  |  |
| MHNO |  |  | Ref. |
| MUNO |  |  | 1.90 (1.77 – 2.05) |
| MHO |  |  | 3.57 (3.08 – 4.15) |
| MUO |  |  | 4.38 (3.93 – 4.88) |
| **Hyperuricemia** |  |  |  |
| MHNO |  |  | Ref. |
| MUNO |  |  | 1.59 (1.45 – 1.73) |
| MHO |  |  | 1.95 (1.67 – 2.27) |
| MUO  CI, confidence interval; HR, hazard ratio; MASLD, metabolic dysfunction  -associated steatotic liver disease; MHNO, metabolically healthy non-obese;  MHO, metabolically healthy obese; MUNO, metabolically unhealthy  non-obese; MUO, metabolically unhealthy obese.  Obesity was defined as BMI ≥25 kg/m² according to the WHO Western  Pacific Regional criteria. |  |  | 2.39 (2.17 – 2.64) |

**Supplementary Table S6** Mediation analysis of metabolic indicators on the association between MHO and MASLD.

| **Mediators** |  | | | | **Total effect**  **HR (95% CI）** | | | |  | **FDR-adjusted *p*-value** | | | | **Indirect effect**  **HR (95% CI）** | | |  | **FDR-adjusted *p*-value** | | **Direct effect**  **HR (95% CI）** | |  | **FDR-adjusted *p*-value** | |  | **Proportion mediated (%)** | |
| --- | --- | --- | --- | --- | --- | --- | --- | --- | --- | --- | --- | --- | --- | --- | --- | --- | --- | --- | --- | --- | --- | --- | --- | --- | --- | --- | --- |
| **Lipid and Glucose Metabolism Group** | | | | | | |  |  |  |  | | | |  | | |  |  | |  | |  |  | |  | |  |
| LDL-C | |  | | | 3.48 (2.97 – 4.06) | | | |  | *p* <0.001 | | | | 1.03 (1.01 – 1.05) | | |  | *p* =0.005 | | 3.36 (2.92 – 3.91) | |  | *p* <0.001 | |  | | 4.8% |
|  | |  | | |  | | | |  |  | | | |  | | |  |  | |  | |  |  | |  | |  |
| TC | |  | | | 3.50 (2.99 – 4.05) | | | |  | *p* <0.001 | | | | 1.01 (0.99 – 1.03) | | |  | *p* =0.195 | | 3.46 (2.98 – 4.01) | |  | *p* <0.001 | |  | | 1.8% |
|  | |  | | |  | | | |  |  | | | |  | | |  |  | |  | |  |  | |  | |  |
| TYG | |  | | | 3.50 (3.01 – 4.07) | | | |  | *p* <0.001 | | | | 1.13 (1.09 – 1.16) | | |  | *p* <0.001 | | 3.09 (2.68 – 3.55) | |  | *p* <0.001 | |  | | 16.2% |
|  | |  | | |  | | | |  |  | | | |  | | |  |  | |  | |  |  | |  | |  |
| RC | |  | | | 3.53 (3.11 – 4.06) | | | |  | *p* <0.001 | | | | 1.03 (1.01 – 1.05) | | |  | *p* =0.006 | | 3.42 (2.96 – 3.94) | |  | *p* <0.001 | |  | | 4.3% |
| **Hepatic Function Group** | | | | | |  |  | | | | | |  | |  |  | | |  |  |  | | |  |  | |  |
| ALT | |  | | | 3.46 (2.99 – 4.01) | | | |  | *p* <0.001 | | | | 1.03 (1.01 – 1.05) | | |  | *p* <0.001 | | 3.37 (2.86 – 3.89) | |  | *p* <0.001 | |  | | 3.5% |
|  | |  | | |  | | | |  |  | | | |  | | |  |  | |  | |  |  | |  | |  |
| AST | |  | | | 3.46 (2.97 – 4.02) | | | |  | *p* <0.001 | | | | 1.00 (0.99 – 1.01) | | |  | *p* =0.508 | | 3.45 (2.97 – 4.01) | |  | *p* <0.001 | |  | | 0.36% |
|  | |  | | |  | | | |  |  | | | |  | | |  |  | |  | |  |  | |  | |  |
| GGT | |  | | | 3.45 (2.95 – 4.01) | | | |  | *p* <0.001 | | | | 1.02 (1.01 – 1.03) | | |  | *p* =0.014 | | 3.40 (2.95 – 3.94) | |  | *p* <0.001 | |  | | 2.0% |
|  | |  | | |  | | | |  |  | | | |  | | |  |  | |  | |  |  | |  | |  |
| ALB | |  | | | 3.46 (2.97 – 4.01) | | | |  | *p* <0.001 | | | | 1.00 (0.99 – 1.01) | | |  | *p* =0.834 | | 3.45 (2.95 – 3.98) | |  | *p* <0.001 | |  | | 0.1% |
|  | |  | | |  | | | |  |  | | | |  | | |  |  | |  | |  |  | |  | |  |
| ALP | |  | | | 3.47 (2.98 – 4.02) | | | |  | *p* <0.001 | | | | 1.01 (0.99 – 1.02) | | |  | *p* =0.167 | | 3.44 (2.95 – 3.97) | |  | *p* <0.001 | |  | | 1.1% |
|  | |  | | |  | | | |  |  | | | |  | | |  |  | |  | |  |  | |  | |  |
| TBIL | |  | | | 3.47 (2.98 – 4.01) | | | |  | *p* <0.001 | | | | 1.01 (1.00 – 1.02) | | |  | *p* =0.010 | | 3.42 (2.95 – 3.97) | |  | *p* <0.001 | |  | | 1.8% |
| **Renal Function Group** | | | | | |  |  | | | | | |  | |  |  | | |  |  |  | | |  |  | |  |
| Scr | |  | | | 3.47 (2.98 – 4.02) | | | |  | *p* <0.001 | | | | 0.99 (0.98 – 1.00) | | |  | *p* =0.272 | | 3.49 (2.98 – 4.02) | |  | *p* <0.001 | |  | | 0.7% |
|  | |  | | |  | | | |  |  | | | |  | | |  |  | |  | |  |  | |  | |  |
| BUN | |  | | | 3.46 (2.97 – 4.01) | | | |  | *p* <0.001 | | | | 1.00 (0.99 – 1.01) | | |  | *p* =0.834 | | 3.46 (2.98 – 4.01) | |  | *p* <0.001 | |  | | 0.1% |
|  | |  | | |  | | | |  |  | | | |  | | |  |  | |  | |  |  | |  | |  |
| UA | |  | | | 3.42 (2.93 – 3.95) | | | |  | *p* <0.001 | | | | 1.07 (1.05 – 1.10) | | |  | *p* <0.001 | | 3.19 (2.69 – 3.70) | |  | *p* <0.001 | |  | | 9.7% |
| **Inflammatory and Immune Response Markers Group** | | | | | | | | | |  |  |  |  |  | | |  |  | |  | |  |  | |  |  | |
| WBC | | | |  | 3.46 (2.96 – 4.03) | | | |  | *p* <0.001 | | | | 1.05 (1.03 – 1.06) | | |  | *p* <0.001 | | 3.29 (2.83 – 3.84) | |  | *p* <0.001 | |  | | 6.7% |
|  | | | |  |  | | | |  |  | | | |  | | |  |  | |  | |  |  | |  | |  |
| Lym Percentage | |  | | | 3.47 (2.97 – 4.04) | | | |  | *p* <0.001 | | | | 1.00 (0.99 – 1.01) | | |  | *p* =0.499 | | 3.46 (2.96 – 4.01) | |  | *p* <0.001 | |  | | 0.4% |
|  | | | |  |  | | | |  |  | | | |  | | |  |  | |  | |  |  | |  | |  |
| Neut Percentage | | |  | | 3.46 (2.96 – 4.03) | | | |  | *p* <0.001 | | | | 1.00 (0.99 – 1.01) | | |  | *p* =0.423 | | 3.45 (2.95 – 4.01) | |  | *p* <0.001 | |  | | 0.4% |
|  | | | |  |  | | | |  |  | | | |  | | |  |  | |  | |  |  | |  | |  |
| SII | | | |  | 3.51 (2.99 – 4.08) | | | |  | *p* <0.001 | | | | 1.02 (1.01 – 1.03) | | |  | *p* =0.056 | | 3.46 (2.97 – 4.01) | |  | *p* <0.001 | |  | | 2.1% |
| **Aging Biomarkers Group** | | | |  |  | | | |  |  | | | |  | | |  |  | |  | |  |  | |  | |  |
| PhenoAgeAccel  ALB, albumin; ALP, alkaline phosphatase; ALT, alanine aminotransferase; AST, aspartate aminotransferase; BUN, blood urea nitrogen; CI, confidence interval; GGT, gamma-glutamyl transferase; HR, hazard ratio; LDL-C, low-density lipoprotein cholesterol; Lym, lymphocyte; MHO, metabolically healthy obese; Neut, neutrophil; RC, remnant cholesterol; Scr, serum creatinine; SII, systemic immune-inflammation index; TBIL, total bilirubin; TC, total cholesterol; TYG, triglyceride-glucose; UA, uric acid; WBC, white blood cell.  All models were adjusted for age and sex.  *p***-**values were adjusted for multiple comparisons using the Benjamini**-**Hochberg procedure (false discovery rate, FDR = 0.05). | | | |  | 3.45 (2.97 – 4.01) | | | |  | *p* <0.001 | | | | 1.02 (1.01 – 1.04) | | |  | *p* =0.017 | | 3.39 (2.90 – 3.91) | |  | *p* <0.001 | |  | | 2.4% |

**Supplementary Table S7** Mediation analysis of metabolic indicators on the association between MHO and hyperuricemia.

| **Mediators** |  | | | | **Total effect**  **HR (95% CI）** | | | |  | **FDR-adjusted *p*-value** | | | | **Indirect effect**  **HR (95% CI）** | | |  | **FDR-adjusted *p*-value** | | **Direct effect**  **HR (95% CI）** | |  | **FDR-adjusted *p*-value** | |  | **Proportion mediated (%)** | |
| --- | --- | --- | --- | --- | --- | --- | --- | --- | --- | --- | --- | --- | --- | --- | --- | --- | --- | --- | --- | --- | --- | --- | --- | --- | --- | --- | --- |
| **Lipid and Glucose Metabolism Group** | | | | | | |  |  |  |  | | | |  | | |  |  | |  | |  |  | |  | |  |
| LDL-C | |  | | | 1.84 (1.55 – 2.12) | | | |  | *p* <0.001 | | | | 1.01 (0.99 – 1.02) | | |  | *p* =0.637 | | 1.82 (1.54 – 2.08) | |  | *p* <0.001 | |  | | 1.6% |
|  | |  | | |  | | | |  |  | | | |  | | |  |  | |  | |  |  | |  | |  |
| TC | |  | | | 1.84 (1.55 – 2.13) | | | |  | *p* <0.001 | | | | 0.99 (0.98 – 1.00) | | |  | *p* =0.694 | | 1.85 (1.55 – 2.12) | |  | *p* <0.001 | |  | | 0.7% |
|  | |  | | |  | | | |  |  | | | |  | | |  |  | |  | |  |  | |  | |  |
| TYG | |  | | | 1.85 (1.55 – 2.13) | | | |  | *p* <0.001 | | | | 1.07 (1.04 – 1.10) | | |  | *p* <0.01 | | 1.73 (1.47 – 1.98) | |  | *p* <0.001 | |  | | 13.8% |
|  | |  | | |  | | | |  |  | | | |  | | |  |  | |  | |  |  | |  | |  |
| RC | |  | | | 1.84 (1.54 – 2.13) | | | |  | *p* <0.001 | | | | 0.99 (0.98 – 1.01) | | |  | *p* =0.694 | | 1.85 (1.57 – 2.11) | |  | *p* <0.001 | |  | | 1.5% |
| **Hepatic Function Group** | | | | | |  |  | | | | | |  | |  |  | | |  |  |  | | |  |  | |  |
| ALT | |  | | | 1.83 (1.53 – 2.12) | | | |  | *p* <0.001 | | | | 1.01 (0.98 – 1.04) | | |  | *p* =0.694 | | 1.81 (1.51 – 2.10) | |  | *p* <0.001 | |  | | 2.9% |
|  | |  | | |  | | | |  |  | | | |  | | |  |  | |  | |  |  | |  | |  |
| AST | |  | | | 1.84 (1.54 – 2.12) | | | |  | *p* <0.001 | | | | 1.00 (0.99 – 1.01) | | |  | *p* =0.973 | | 1.84 (1.54 – 2.09) | |  | *p* <0.001 | |  | | 0.35% |
|  | |  | | |  | | | |  |  | | | |  | | |  |  | |  | |  |  | |  | |  |
| GGT | |  | | | 1.82 (1.53 – 2.11) | | | |  | *p* <0.001 | | | | 1.02 (1.01 – 1.03) | | |  | *p* =0.048 | | 1.79 (1.53 – 2.05) | |  | *p* <0.001 | |  | | 3.7% |
|  | |  | | |  | | | |  |  | | | |  | | |  |  | |  | |  |  | |  | |  |
| ALB | |  | | | 1.84 (1.55 – 2.13) | | | |  | *p* <0.001 | | | | 1.02 (1.01 – 1.03) | | |  | *p* =0.021 | | 1.81 (1.54 – 2.06) | |  | *p* <0.001 | |  | | 3.7% |
|  | |  | | |  | | | |  |  | | | |  | | |  |  | |  | |  |  | |  | |  |
| ALP | |  | | | 1.84 (1.54 – 2.13) | | | |  | *p* <0.001 | | | | 1.00 (0.99 – 1.01) | | |  | *p* =0.994 | | 1.84 (1.55 – 2.11) | |  | *p* <0.001 | |  | | 0.04% |
|  | |  | | |  | | | |  |  | | | |  | | |  |  | |  | |  |  | |  | |  |
| TBIL | |  | | | 1.85 (1.55 – 2.16) | | | |  | *p* <0.001 | | | | 1.00 (0.99 – 1.01) | | |  | *p* =0.671 | | 1.84 (1.56 – 2.13) | |  | *p* <0.001 | |  | | 1.1% |
| **Renal Function Group** | | | | | |  |  | | | | | |  | |  |  | | |  |  |  | | |  |  | |  |
| Scr | |  | | | 1.83 (1.54 – 2.12) | | | |  | *p* <0.001 | | | | 1.01 (0.99 – 1.02) | | |  | *p* =0.694 | | 1.82 (1.54 – 2.08) | |  | *p* <0.001 | |  | | 1.1% |
|  | |  | | |  | | | |  |  | | | |  | | |  |  | |  | |  |  | |  | |  |
| BUN | |  | | | 1.84 (1.55 – 2.13) | | | |  | *p* <0.001 | | | | 1.00 (0.99 – 1.01) | | |  | *p* =0.861 | | 1.84 (1.55 – 2.13) | |  | *p* <0.001 | |  | | 0.27% |
| **Inflammatory and Immune Response Markers Group** | | | | | | | | | |  |  |  |  |  | | |  |  | |  | |  |  | |  |  | |
| WBC | | | |  | 1.84 (1.55 – 2.13) | | | |  | *p* <0.001 | | | | 1.02 (1.01 – 1.04） | | |  | *p* =0.021 | | 1.80 (1.51 – 2.08) | |  | *p* <0.001 | |  | | 5.5% |
|  | | | |  |  | | | |  |  | | | |  | | |  |  | |  | |  |  | |  | |  |
| Lym Percentage | |  | | | 1.84 (1.55 – 2.13) | | | |  | *p* <0.001 | | | | 1.00 (0.99 – 1.00) | | |  | *p* =0.973 | | 1.84 (1.55 – 2.11) | |  | *p* <0.001 | |  | | 0.02% |
|  | | | |  |  | | | |  |  | | | |  | | |  |  | |  | |  |  | |  | |  |
| Neut Percentage | | |  | | 1.84 (1.55 – 2.13) | | | |  | *p* <0.001 | | | | 1.00 (0.99 – 1.00) | | |  | *p* =0.973 | | 1.84 (1.55 – 2.13) | |  | *p* <0.001 | |  | | 0.02% |
|  | | | |  |  | | | |  |  | | | |  | | |  |  | |  | |  |  | |  | |  |
| SII | | | |  | 1.84 (1.55 – 2.13) | | | |  | *p* <0.001 | | | | 1.00 (0.99 – 1.00) | | |  | *p* =0.868 | | 1.84 (1.55 – 2.13) | |  | *p* <0.001 | |  | | 0.03% |
| **Aging Biomarkers Group** | | | |  |  | | | |  |  | | | |  | | |  |  | |  | |  |  | |  | |  |
| PhenoAgeAccel  ALB, albumin; ALP, alkaline phosphatase; ALT, alanine aminotransferase; AST, aspartate aminotransferase; BUN, blood urea nitrogen; CI, confidence interval; GGT, gamma-glutamyl transferase; HR, hazard ratio; LDL-C, low-density lipoprotein cholesterol; Lym, lymphocyte; MHO, metabolically healthy obese; Neut, neutrophil; RC, remnant cholesterol; Scr, serum creatinine; SII, systemic immune-inflammation index; TBIL, total bilirubin; TC, total cholesterol; TyG, triglyceride-glucose; WBC, white blood cell.  All models were adjusted for age and sex.  *p***-**values were adjusted for multiple comparisons using the Benjamini*-*Hochberg procedure (false discovery rate, FDR = 0.05). | | | |  | 1.80 (1.51 – 2.09) | | | |  | *p* <0.001 | | | | 0.98 (0.97 – 1.00) | | |  | *P =*0.231 | | 1.83 (1.54 – 2.11) | |  | *p* <0.001 | |  | | 3.0% |
